# Menstrual Effluent Provides a Novel Diagnostic Window on the Pathogenesis of Endometriosis

**DOI:** 10.3389/frph.2020.00003

**Published:** 2020-07-22

**Authors:** Ashima Nayyar, Matthew I. Saleem, Mine Yilmaz, Margaret DeFranco, Gila Klein, Kristine Mae Elmaliki, Elena Kowalsky, Prodyot K. Chatterjee, Xiangying Xue, Radhika Viswanathan, Andrew J. Shih, Peter K. Gregersen, Christine N. Metz

**Affiliations:** ^1^Feinstein Institutes for Medical Research, Northwell Health, Manhasset, NY, United States; ^2^Donald and Barbara Zucker School of Medicine at Hofstra/Northwell, Hempstead, NY, United States

**Keywords:** ALDH1A1, endometriosis, endometrium, inflammatory cytokines, non-invasive diagnostic, podoplanin, stromal fibroblast cells

## Abstract

Endometriosis is a chronic inflammatory disorder characterized by the presence of endometrial-like tissue growing outside of the uterus. Although the cause is unknown, retrograde menstruation leads to deposition of endometrial cells into the peritoneal cavity. Lack of disease recognition and long diagnostic delays (6–10 years) lead to substantial personal, social and financial burdens, as well as delayed treatment. A non-invasive diagnostic for endometriosis is a major unmet clinical need. Here, we assessed whether differences in menstrual effluent-derived stromal fibroblast cells (ME-SFCs) from women with and without endometriosis provide the basis for a non-invasive diagnostic for endometriosis. In addition, we investigated whether treatment of control ME-SFCs with inflammatory cytokines (TNF and IL-1β) could induce an endometriosis-like phenotype. ME-SFCs from laparoscopically diagnosed endometriosis patients exhibit reduced decidualization capacity, measured by IGFBP1 production after exposure to cAMP. A receiver operating characteristic (ROC) curve developed using decidualization data from controls and endometriosis subjects yielded an area under the curve of 0.92. In addition, a significant reduction in *ALDH1A1* gene expression and increased podoplanin surface expression were also observed in endometriosis ME-SFCs when compared to control ME-SFCs. These endometriosis-like phenotypes can be reproduced in control ME-SFCs by exposure to inflammatory cytokines (TNF and IL-1β) and are associated with increased cell migration. These results are consistent with the hypothesis that chronic intrauterine inflammation influences the development of endometriosis lesions following retrograde menstruation. In conclusion, the analysis of ME-SFCs can provide an accurate, rapid, and non-invasive diagnostic for endometriosis and insight into disease pathogenesis.

## Introduction

Endometriosis is a common, complex, and chronic inflammatory disorder associated with debilitating pelvic pain, dysmenorrhea, and infertility (1, 2). It is defined by the growth of endometrial-like tissues containing epithelial glands and stromal cells outside of the uterus, primarily in the peritoneal cavity. Although the exact cause(s) of endometriosis are not known, a commonly accepted theory is based on a causative role of retrograde menstruation (3), whereby endometrial tissue containing stromal cells is shed and delivered to the peritoneal cavity. Consistent with this theory, spontaneous endometriosis is only observed in the few animals that menstruate (4) and endometriosis can be further induced by injecting shed menstrual tissues into recipient non-human primates (5, 6).

In addition to the well-documented inflammation associated with ectopic endometriosis lesions (7–11), significant inflammation is reported in the eutopic endometrium of women with endometriosis (9, 12–14). Specifically, increased endometrial TNF, IL-1β, and CCL17 expression are observed in endometriosis (13, 15, 16). Furthermore, chronic endometritis, a poorly diagnosed condition characterized by persistent endometrial inflammation, is a significant risk factor for endometriosis (17, 18) and highlights the potential role of chronic endometrial inflammation in endometriosis.

One of the most challenging problems for patients with endometriosis is its diagnosis. Currently, diagnosis requires invasive laparoscopic surgery and typically takes 6–10 years from the onset of symptoms to diagnosis (2, 19, 20). This delay is costly on many levels, including loss of productivity, poor quality of life, and extensive and unproductive use of medical services, as well as increased infertility (20). Consistent with the differences in the eutopic endometrium of women with and without endometriosis, we have previously shown distinct differences in menstrual effluent (ME) and menstrual effluent-derived stromal fibroblast cells (ME-SFCs) obtained from a small cohort of women with endometriosis compared to healthy controls, including impaired stromal cell decidualization and reduced expression of *ALDH1A1* and other genes associated with the retinoic acid pathway (21). These findings support the development of an ME-based non-invasive diagnostic, a serious unmet clinical need.

ME also offers a window for investigating uterine abnormalities involved in the causative pathways for endometriosis. Despite several reports implicating chronic endometritis and persistent endometrial inflammation in the pathogenesis of endometriosis (17, 18, 22, 23), most endometriosis research to date has focused on inflammation associated with ectopic lesions typically found in the pelvic cavity and surgical specimens [Reviewed in (2, 24–26)]. The availability of ME, which can be easily collected in a relatively non-invasive manner, provides a biologic resource to examine the effects of inflammation on ME-derived cells, namely endometrial stromal cells, which are found in the lesions. Here, we also investigated whether exposure of control ME-SFCs to chronic inflammation *ex vivo* would recapitulate the endometriosis-like phenotype and lead to sustained endometriosis-like cellular alterations in these cells.

## Materials and Methods

### Menstrual Effluent Samples and Subject Details

Women of reproductive age (24–49 years) who were not pregnant or breastfeeding, who were menstruating and willing to provide menstrual effluent (ME) samples were recruited and consented. Women with histologically confirmed endometriosis (determined following laparoscopic surgery and documented in a pathology report) were recruited and enrolled as “endometriosis” subjects through the ROSE study (https://feinstein.northwell.edu/institutes-researchers/institute-molecular-medicine/robert-s-boas-center-for-genomics-and-human-genetics/rose-research-outsmarts-endometriosis). Women who self-reported symptoms consistent with endometriosis (e.g., recurrent dysmenorrhea; dyspareunia; dysuria; dyschezia; and/or persistent abdominal bloating), but have not yet been diagnosed with endometriosis were recruited and enrolled as “symptomatic” subjects through the ROSE study. Control subjects who self-reported no history suggestive of a diagnosis of endometriosis were recruited and enrolled through the GaP registry (https://feinstein.northwell.edu/institutes-researchers/institute-molecular-medicine/robert-s-boas-center-for-genomics-and-human-genetics/gap-registry). For each set of experiments, endometriosis subjects (cases) and control subjects were age-matched within 5–6 years of age (see Table 1). Note: formal sample size calculations were not performed, as the study of menstrual effluent-derived stromal cells does not involve ethical, time or cost issues [which warrant sample size calculations (27)] and the approach to using ME is novel, with limited available data. Sample sizes for the decidualization assays and *ALDH1A1* mRNA expression analyses were based on our prior studies (21). For IGFBP1, with an estimated effect size of 1.42 we rejected the null hypothesis at *p* = 0.03 with *n* = 7 endometriosis cases and *n* = 7 controls (21); the sample size for this study was tripled. For *ALDH1A1* mRNA expression, with an estimated effect size of 1.09 we rejected the null hypothesis at *p* = 0.04 with *n* = 7 cases and n = 7 controls (21); the sample size for this study was more than tripled. Since this is the first report to assess podoplanin (PDPN) surface expression by ME-SFCs, no sample size calculations were performed. The results of this study will inform the design of future clinical studies to incorporate PDPN expression, as well as *ALDH1A1* mRNA expression into a multivariate diagnostic test to improve the specificity of the diagnostic based on IGFBP1 analysis.

**Table 1:** Subject and cell passage information, sample sizes and statistics used for all experiments.

**Expt**	**Group**	**Sample size (*n*)**	**Assay**	**Passage number**	**Age (years) mean ± SD**		**Statistical analyses**
I	CTRL	23	Decidualization	0	34.3 ± 6.8	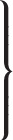	Kruskal-Wallis with
	ENDO	24	Decidualization	0	37.3 ± 4.3		Dunn's Multiple
	SYMPTO	9	Decidualization	0	31.3 ± 5.2		Comparison
II	CTRL	34	*ALDH1A1* mRNA	0	33.8 ± 6.8	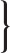	Unpaired students *t*-test
	ENDO	30	*ALDH1A1* mRNA	0	37.4 ± 4.2		with Welch's correction
III	CTRL	7	PDPN	1	33.6 ± 4.2	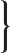	Unpaired students *t*-test
	ENDO	7	PDPN	1	34.9 ± 1.3		with Welch's correction
IV	CTRL ± TNF	5	Decidualization	0	30.2 ± 3.2		Paired *t*-test
V	CTRL ± TNF	19	*ALDH1A1* mRNA	0	31.9 ± 6.0		Paired *t*-test
VI	CTRL ± TNF	9	PDPN	1	33.6 ± 7.9		Paired *t*-test
VII	CTRL	15	Cell migration	2	37.1 ± 9.3	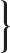	Unpaired students *t*-test
	ENDO	16	Cell migration	2	34.1 ± 5.6		with Welch's correction
VIII	CTRL ± TNF	6	Cell migration	2	33.5 ± 7.5		Paired *t*-test
IX	CTRL	12	Cell adhesion	1	32.8 ± 6.5	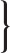	Unpaired students *t*-test
	ENDO	9	Cell adhesion	1	37.6 ± 6.6		with Welch's correction
X	CTRL ± TNF	9	Decidualization over 1–3 weeks	1	34.4 ± 7.2		Repeated measures ANOVA
XI	CTRL ± IL-1β	8	Decidualization over 1–3 weeks	1	33.4 ± 7.3		Repeated measures ANOVA

*CTRL, Control subjects; ENDO, endometriosis subjects; SYMPTO, symptomatic subjects. No groups (CTRL vs. ENDO or CTRL vs. SYMPTO) were significantly different with respect to age*.

### Collection, Isolation, and Culture of Menstrual Effluent-Derived Stromal Fibroblasts Cells (ME-SFCs)

Subjects collected their ME for 4–8 h on the day of their heaviest menstrual flow (typically day 1 or 2 of the cycle) using either a menstrual cup or a novel menstrual collection sponge. After collection, they shipped their ME at 4°C to the laboratory for processing. For saturated menstrual collection sponges, ME was collected by rinsing the sponges with 1X PBS (Gibco/Thermo Fisher Scientific, Waltham, MA, US), followed by a brief trypsinization using Trypsin-EDTA (Gibco/Thermo Fisher) at 37°C/5%CO2; ME cells were collected after a brief centrifugation (×300 g) and resuspended in 1 ml growth media comprised of DMEM (Gibco/Thermo Fisher) containing 10% fetal bovine serum (FBS, mesenchymal stem cell qualified) (Gibco/Thermo Fisher), 1% penicillin/streptomycin (Gibco/Thermo Fisher), 1% L-glutamine (Gibco) and Normocin (1:500) (Invivogen, San Diego, CA, US). Approximately 500 μl of resuspended ME was plated per T-75 flask in growth media. ME collected from menstrual cups was plated directly in growth media (500 μl per T-75 flask). After a 24 h incubation at 37°C/5%CO_2_, flasks were aspirated and washed with 1X PBS and then growth media was replaced. ME-SFC cultures were monitored over time by visualization under a light microscope and growth media was replaced every 3–4 days. ME-SFCs were passaged 1:6 after brief trypsinization. Flow cytometry studies show that ME-SFCs are >98% pure, as determined by CD45^−^/CD73^+^/CD90^+^/CD105^+^ staining (21). The investigators introduced the novel menstrual collection sponge to eliminate the barrier to participation due to the menstrual cup. Optimization studies for ME-SFCs isolated from menstrual sponges and menstrual cups (from the same subjects collected on the same day) showed that resultant ME-SFCs exhibited similar purity and staining by flow cytometry, comparable growth patterns, and nearly identical decidualization capacity. Therefore, ME-SFC preparations from cups and sponges were considered equivalent. All experiments were initiated using confluent low passage ME-SFC monolayers (passages 0–2 [p0-p2]), as indicated. For each experimental study, ME-SFCs were exactly matched on cell passage number and age-matched within 5–6 years (see Table 1).

### Decidualization Assays

Decidualization assays were initiated using p0 ME-SFCs (*n* = 23 controls, *n* = 24 endometriosis subjects, and *n* = 9 symptomatic subjects). For each subject, 1.5 × 10^4^ ME-SFCs in 200 μl of growth media (as described above) were plated in a 96 well plate and allowed to grow until they were confluent (~2–3 days). Confluent ME-SFCs were incubated at 37°C/5%CO_2_ in decidualization media (growth media containing 2% FBS, instead of 10%FBS) and treated with either 0.5mM 8-Bromoadenosine 3′,5′-cyclic monophosphate sodium salt (cAMP) (Sigma-Aldrich, St. Louis, MO, US) (*n* = 3 wells per subject) or vehicle (1X PBS, *n* = 3 wells per subject). After 24 h culture supernatants were collected after a brief centrifugation and cell-free supernatants were analyzed for IGFBP1 concentrations by ELISA using R&D Systems™ Human IGFBP1 DuoSet (R&D Systems®, Minneapolis, MN, US) according to the manufacturer's directions and similar to that described in Warren et al. (21). For the ELISA, vehicle-supernatants were diluted 1:2 and 1:4 and cAMP-supernatants were diluted 1:50–1:250 (these dilutions ensured that IGFBP1 concentrations were within the linear range of the standard curve for quantification). All standards and samples were tested in triplicate (technical replicates) by ELISA following the manufacturer's instructions. Immediately after adding the stop solution (Fisher Scientific), optical densities/absorbance readings were obtained at 450 nm/570 nm using an ELISA plate reader (MRX plate reader, Dynex/Dynatech). The average of the triplicate readings was used to estimate the concentration using the IGFBP1 standard curve. The average coefficient of variation (%CV) for individuals' triplicate samples for the IGFBP1 ELISA was 5.5 (similar to the manufacturer's published %CV = 6, range 0.8–14.1). After determining the “Veh” and “cAMP” protein values for each pair of wells, the formula: ratio of cAMP (IGFBP1): vehicle (IGFBP1) was used to determine the induction of IGFBP1 by cAMP (i.e., decidualization capacity).

For examining the effects of chronic TNF or IL-1β on ME-SFC decidualization capacity, ME-SFCs (p0) (*n* = 5 control subjects) were treated with vehicle or recombinant TNF [purified as described in (28)] (10 ng/ml) on days 1 and 3. On day 7, ME-SFCs were lifted after a brief trypsinization and plated for assessment of decidualization capacity, as described above. In a separate experiment ME-SFCs (p1) from 8 to 9 control subjects were treated with either TNF (1ng/ml) or IL-1β (PeproTech Inc., Rocky Hill, NJ, US) (1 ng/ml) on days 1 and 3 in growth media; cells were then cultured in growth media alone (without cytokines) and on day 7 (1 week), day 14 (2 weeks), and day 21 (3 weeks) cultures were tested for decidualization capacity (as described above).

### Determination of *ALDH1A1* mRNA Expression by ME-SFCs

#### ME-SFC Culture Conditions

For quantifying the differences in *ALDH1A1* mRNA expression, p0 ME-SFCs (*n* = 34 controls and *n* = 30 endometriosis cases) were plated in growth media (as described above) at 1 × 10^4^ cells per well (using a 6 well plate) (3 wells per subject, i.e., technical replicates). ME-SFCs were grown to confluency and then growth media was replaced with decidualization media (as described above) and incubated for 24 h.

#### RNA Extraction and cDNA Synthesis

ME-SFCs were lifted using trypsin, collected in RLT buffer with 2-mercaptoethanol (2-ME) and stored at −80°C. RNA was purified using the RNeasy plus mini kit (Qiagen, Germantown, MD, US) and Qiashredder (Qiagen) according to the manufacturer's suggestions. RNA concentration and quality were determined by Nanodrop (Fisher Scientific). Total RNA (500 ng) was converted to cDNA using the SuperScript™ IV VILO™ cDNA Synthesis Kit (Thermo Fisher) in Applied Biosystems Veriti Thermal cycler.

#### Quantitative RT PCR

Real time quantitative RT-PCR was carried out with TaqMan™ Gene Expression Master Mix (Thermo Fisher Scientific) using the Viaa7 Flex Real-Time PCR System (Thermo Fisher Scientific) in 10 μl reaction volumes using 384 well plates. Taqman assays were used for *ALDH1A1* (Thermo Fisher Scientific) and *HPRT1* (Thermo Fisher Scientific). All reactions were carried out in triplicate and fold-change expression values were averaged. Relative difference in the gene expression was normalized to expression levels of a housekeeping gene, *HPRT1*. The average %CV (for individuals' triplicate samples) for *ALDH1A1* mRNA expression analyzed by real time qPCR was 0.50 (range 0.05–2.0, based on Ct values).

For examining the effects of chronic TNF on *ALDH1A1* mRNA expression, ME-SFCs (p0) (*n* = 19 control subjects) were treated with vehicle or TNF (10 ng/ml) on days 1 and 3. On day 7, ME-SFCs were lifted after a brief trypsinization and plated for assessment of *ALDH1A1* mRNA expression, as described above.

### Cell Migration Assays

Cell migration assays were performed using p2 ME-SFCs (*n* = 15 controls; *n* = 16 endometriosis patients), using the scratch assay as previously described (29, 30). Briefly, confluent ME-SFCs were lifted using trypsin and plated at 1 × 10^5^ cells/ml (700 μl/well in a 24 well plate) in growth media (*n* = 3 wells for each subject's ME-SFCs). Once the ME-SFCs were at least 90% confluent, one vertical and one horizontal scratch was made using a sterile 200 μl pipette tip; cell debris were aspirated, cells were washed and the media was replaced with decidualization media containing mitomycin C (Fisher Scientific) (5 μg/ml final, to prevent cell proliferation). Images were taken on the Zeiss Axiovert 200M Apotome Microscope (Zeiss) at time 0 and 20 h later at 10X (4 images per well × 3 wells per sample). The 20-h time point was chosen because optimization studies showed that the average % wound closure with control ME-SFCs was ~50% at 20 h; thus, an increase (or decrease in cell migration) could be detected. Images were imported into Image J and % wound closure was calculated as described by Venter and Niesler (29).

For examining the effects of chronic TNF on cell migration, p2 ME-SFCs were treated with vehicle or TNF (10 ng/ml) on days 1 and 3 (*n* = 6 controls); on day 7 ME-SFCs were lifted with trypsin and then used for migration assays, as described above.

### Adhesion Assays

Cell adhesion was determined using p1 ME-SFCs from control (*n* = 12) and endometriosis (*n* = 9) subjects. Confluent ME-SFCs were lifted with ACCUTASE™ (BD Biosciences, San Jose, CA, US) and resuspended in serum-free growth media. Cells were plated in two sets of 96 well plates, one plated with the total cell number “total” plate and the other plated for the adherent cell number “adherent plate”. Adherent plates were coated with fibronectin (Fisher Scientific) (5 μg/ml, 50 μl/well), covered and kept overnight at room temperature on the shaker. Fibronectin was chosen as an extracellular matrix substrate because increased fibronectin production by peritoneal macrophages in endometriosis has been reported (31). The plates were washed with PBS to remove excess fibronectin and next day 2 × 10^4^ ME-SFCs per well was plated in triplicate for each subject on each plate. In addition, a standard curve was determined after plating: 2 × 10^4^, 1 × 10^4^, 0.5 × 10^4^, 0.1 × 10^4^ ME-SFCs per well in serum-free growth media and media alone or no cells per well (in triplicate) in “total” plate. The “adherent” and “total” plates were incubated for 20 min and 2 h (for maximum adherence and determination of total cell number), respectively, at 37°C and 5% CO_2_. After incubation, the “adherent” plate was turned upside down on paper towels, patted three times, and gently washed twice with PBS to remove loosely attached cells. Note: the “adherent” plate was not centrifuged. The “total plate” was centrifuged at 300 × g for 5 min and the supernatant was gently aspirated. Both plates were frozen at −80°C. The next day the plates were analyzed using the CyQUANT assay (Fisher Scientific) to determine the number of cells per well, according to manufacturer's guidelines. Briefly, frozen plates were thawed and lysed by the addition of a buffer containing the CyQUANT GR dye; the wells were incubated for 10 min and fluorescence was then measured in the Synergy H1Hybrid Multi-Mode Reader (BioTek) at 480 nm excitation and 520 nm emission. A standard curve was plotted for cell number vs. fluorescence. The percentage adhesion was calculated as the ratio of the difference between the total cells and adherent cells over total number of cells multiplied by 100 (23).


$$\begin{array}{l} \text{Adhesion }\%=\frac{Total\;cell\;number-Adherent\;cell\;number}{Total\;cell\;number}\times \;100 \end{array}$$

### Analysis of Cell Surface Podoplanin Expression by Flow Cytometry

Confluent monolayers of p1 ME-SFCs from endometriosis (*n* = 7) and control (*n* = 7) subjects were lifted with ACCUTASE™, centrifuged (×300 g) and resuspended in staining buffer (PBS containing 1% FBS, 0.1% azide) with either antibodies against BV421-CD73 (BioLegend, San Diego, CA, US) and PE/Cy7-podoplanin (PDPN) (BioLegend) or appropriate isotype control antibodies BV421-Mouse IgG1, κ (BioLegend) and PE/Cy7-Rat IgG2a, κ (BioLegend) for 30 min at 4°C in the dark; the cells were washed twice with 1%FBS in PBS and fixed with 1.5% buffered formalin. Zombie Red™ Fixable Viability Kit (BioLegend) was used to determine live/dead populations. All data were collected on the Fortessa Flow Cytometer (BD, Franklin lakes, NJ, US) and analyzed using FlowJo software (version 10.1r5) (FLOWJO LLC, https://www.flowjo.com/) to determine geometric mean MFI (corrected for isotype control).

For examining the effect of TNF on PDPN expression, p1 ME-SFCs were treated with vehicle or TNF (1 or 10 ng/ml) for 48 h (*n* = 9 controls) in separate flasks, and then analyzed for PDPN surface expression by flow cytometry as described above.

### Statistical Analysis

GraphPad Prism version 5.0 (GraphPad Software Inc., http://www.graphpad.com/scientific-software/prism/) was used for all statistical analyses. Statistical parameters, including the type of tests, number of samples (n), descriptive statistics and significance are reported in the figures, figure legends and Table 1. Different statistical models were employed for various experiments; the appropriate statistical assumptions were met for statistical analyses (see Table 1). For decidualization studies comparing endometriosis, control, and symptomatic subjects, the Kruskal-Wallis test with Dunn's multiple comparisons *post hoc* test was employed. The ROC curve was determined as described by Goksuluk et al. (32). Paired *t*-tests were used for all assays (decidualization, *ALDH1A1* mRNA expression, cell migration, and PDPN expression) comparing control ME-SFC responses in the presence and absence of chronic TNF exposure when two groups were compared. Unpaired *t*-tests with Welch's correction (for unequal variances) were used when comparing ME-SFCs from endometriosis cases and controls for *ALDH1A1* expression, cell migration, and PDPN expression. Repeated measures ANOVA (using natural log-transformed data; TNF/Vehicle or IL-1β/Vehicle) was used to analyze the effect of TNF or IL-1β on decidualization over weeks 1–3 (**Figure 6** and Supplemental Figures 3A–D). Once it demonstrated that the patterns were the same across time (weeks 1–3), i.e., there was no difference in effect over time, then the means at each week were pooled and a one sample *t*-test was used for comparison. *P* < 0.05 were considered significant.

## Results

### Three Distinct Phenotypes Distinguish ME-SFCs From Endometriosis Patients vs. Healthy Controls—Decidualization, *ALDH1A1* Gene Expression and Podoplanin Surface Expression

**Figure 1:**
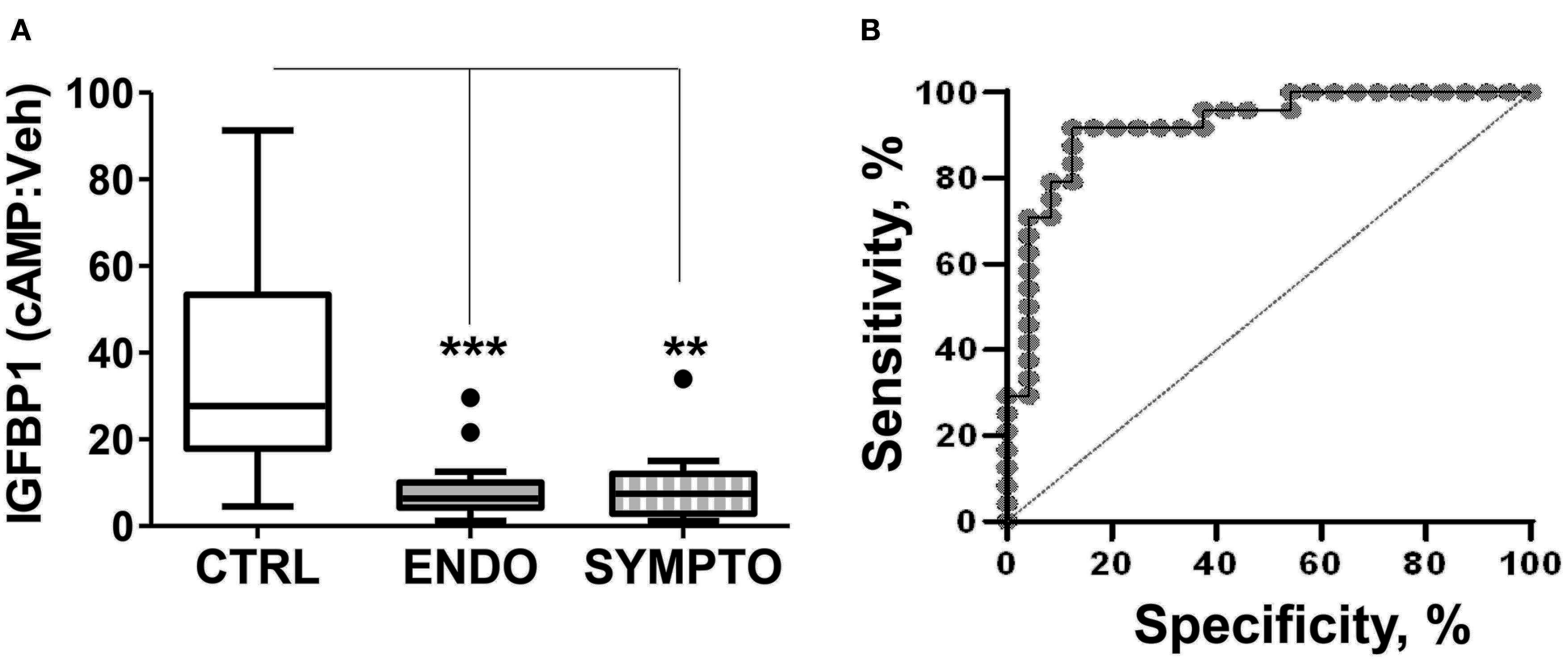
ME-SFCs from endometriosis patients and symptomatic, undiagnosed patients exhibit defective decidualization. **(A)** ME-SFCs cultured from healthy controls (CTRL, *n* = 23), surgically diagnosed endometriosis patients (ENDO, *n* = 24) and women with symptoms suggestive of endometriosis, but not diagnosed (SYMPTO, *n* = 9) were treated with vehicle and cAMP (0.5 mM) in decidualization media. After 24 h, decidualization was determined by measuring IGFBP1 concentrations in the culture supernatants by ELISA. Decidualization capacity for healthy controls (white box) and endometriosis cases (gray box) are shown as the ratio of cAMP-IGFBP1:vehicle-IGFBP1 using Tukey box and whisker plots (box = interquartile range [25th and 75th percentile]; horizontal line = median; upper and lower whiskers indicate range without outliers; outliers = •). Significance was determined using the Kruskal-Wallis test followed by Dunn's Multiple Comparisons *post hoc* comparison. **P < 0.01; ***P < 0.001. **(B)** Receiving operator characteristic curve or ROC curve shows the visual representation of the decidualization (IGFBP1) data for the healthy controls and the surgically diagnosed endometriosis patients in **(A)**. The area under the curve (AUC) = 0.92.

We compared the decidualization capacity of ME-SFCs obtained from a cohort of surgically diagnosed endometriosis subjects (*n* = 24) and age-matched control subjects (*n* = 23, without endometriosis symptoms). As shown, in Figure 1A, ME-SFCs from endometriosis subjects exhibit a dramatic and highly significant reduction in decidualization, as determined by measuring the production of IGFBP1 in the culture supernatants by ELISA (*P* < 0.001). Similarly, ME-SFCs from symptomatic patients (*n* = 9) exhibit a similar defect in their decidualization capacity when compared to healthy controls (Figure 1A, *P* < 0.01). A receiver operating characteristic (ROC) curve was developed using the IGFBP1 data from controls and endometriosis subjects with a confirmed surgical diagnosis from Figure 1A yielding an area under the curve (AUC) of 0.92 (Figure 1B), with a *p*-value of 1.3E^−25^. The optimal cut-off point was 14.21 for IGFBP1 by ELISA, as determined by (32); this was identical to the optimal cut-off determined using the Youden Index (J) method. The sensitivity was 0.875 (lower limit = 0.676 and upper limit = 0.973) and the specificity was 0.917 (lower limit = 0.73 and upper limit = 0.99). These data support the utility of an ME-SFCs-based decidualization assay as a potential non-invasive diagnostic for endometriosis.

**Figure 2:**
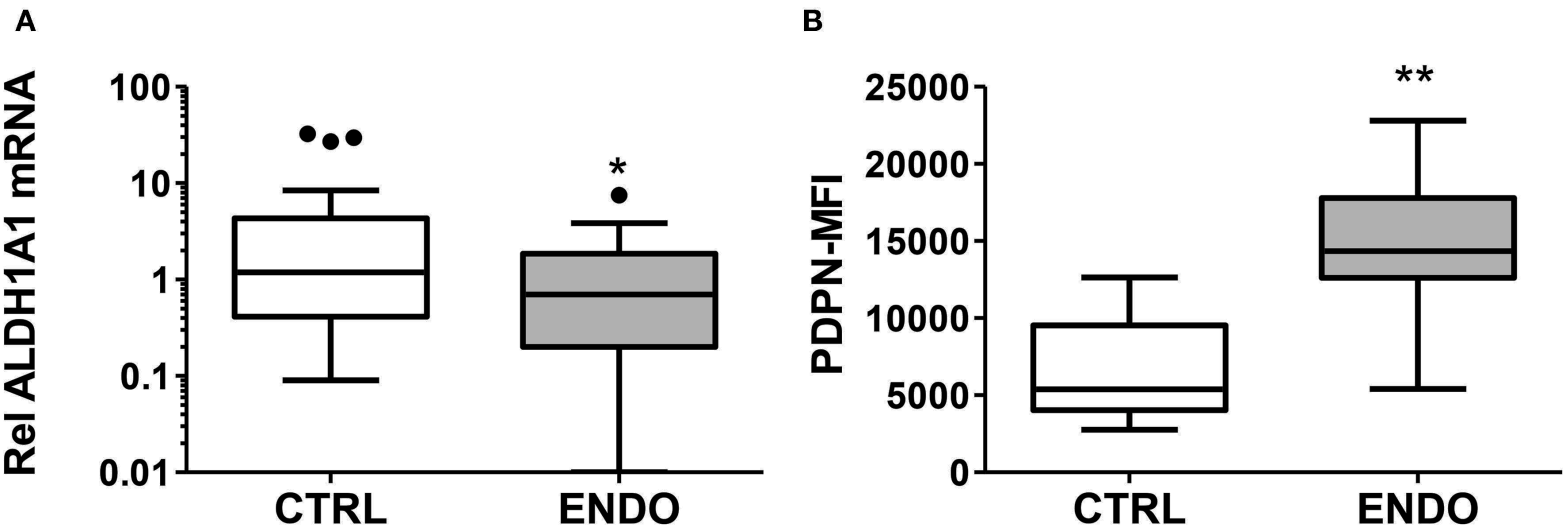
ALDH1A1 gene expression is reduced and PDPN surface expression is increased by ME-SFCs from endometriosis patients. **(A)** *ALDH1A1* mRNA expression. Confluent monolayers of ME-SFCs from healthy controls (CTRL, *n* = 34) and surgically diagnosed endometriosis patients (ENDO, *n* = 30) were incubated decidualization media for 24 h and then analyzed for *ALDH1A1* mRNA expression by RT-qPCR. Relative differences in gene expression for each subject were normalized to expression levels of a housekeeping gene, *HPRT1*. Data are shown for controls (white box) and endometriosis cases (gray box) using Tukey box and whisker plots (box = interquartile range; horizontal line = median; upper and lower whiskers indicate range without outliers; outliers = •). **(B)** Podoplanin (PDPN) surface expression. Confluent monolayers of ME-SFCs from healthy controls (CTRL, *n* = 7) and surgically diagnosed endometriosis patients (ENDO, *n* = 7) were analyzed in duplicate for PDPN surface expression by flow cytometry. MFI data (corrected for isotype control) are shown for controls (white boxes) and endometriosis subjects (gray box) using Tukey box and whisker plots (box = interquartile range; horizontal line = median; upper and lower whiskers indicate range without outliers; outliers = •). Significance was determined using unpaired students *t*-test with Welch's correction for unequal variances. *P < 0.05; **P < 0.01.

In order to explore other stromal cell phenotypes useful for more accurately diagnosing endometriosis, we examined *ALDH1A1* gene expression comparing cells from endometriosis patients with cells from healthy controls. As shown in Figure 2A, we have confirmed in a larger cohort (*n* = 34 controls and *n* = 30 endometriosis patients) the significant reduction in *ALDH1A1* mRNA expression in ME-SFCs from endometriosis patients compared with cells from controls (*P* = 0.016).

In addition, given the invasive pathologic appearance of endometriosis lesions containing stromal fibroblast cells, we searched for evidence of changes in ME-SFCs that might correlate with an invasive and/or inflammatory phenotype. As shown in Figure 2B, ME-SFCs cultured from endometriosis patients exhibit increased expression of PDPN on their surface when compared to ME-SFCs from controls (*P* < 0.01).

### Exposure of ME-SFCs to Inflammatory Cytokines Induces an Endometriosis-Like Phenotype

**Figure 3:**
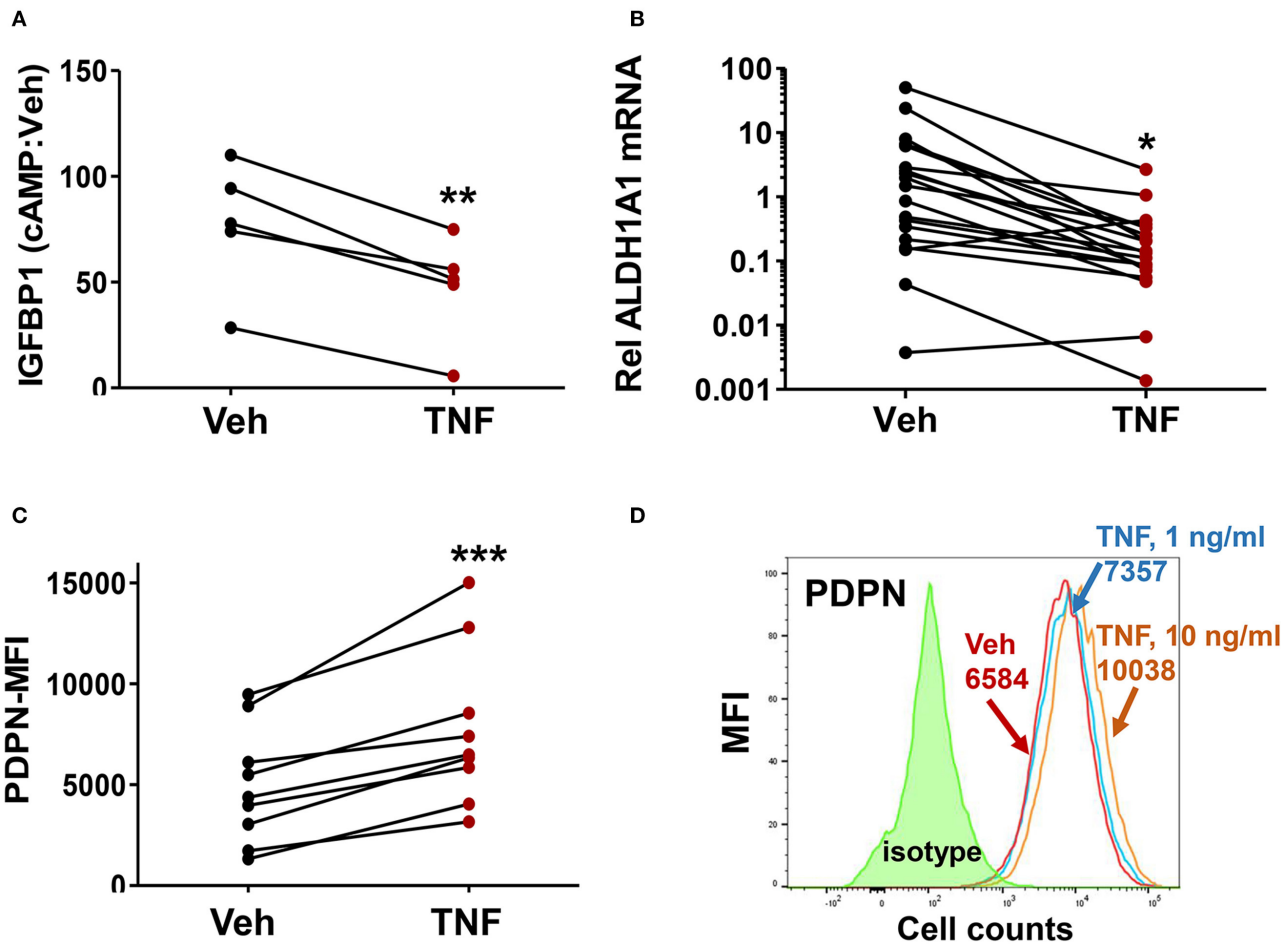
Treatment of control ME-SFCs with TNF induces an endometriosis-like phenotype. **(A)** Inhibition of IGFBP1 by TNF. Healthy control ME-SFCs (*n* = 5) were treated with vehicle (Veh) and TNF (10 ng/ml) in separate flasks on days 1 and 3. On day 7 ME-SFCs were lifted, washed and plated in decidualization media. Decidualization capacity was analyzed after treating the vehicle- and TNF-treated cells with vehicle and cAMP (0.5 mM), as described in Figure 1A. After 24 h culture supernatants were analyzed for IGFBP1 concentrations by ELISA. Decidualization capacity (the ratio of cAMP-IGFBP1:vehicle-IGFBP1) for each subject under both conditions was determined as in Figure 1A. **(B)** Inhibition of *ALDH1A1* mRNA expression by TNF. Healthy control ME-SFCs (*n* = 19) were treated with vehicle (Veh) and TNF (10 ng/ml), in separate flasks, on days 1 and 3. On day 7 cells were lifted, washed and plated in decidualization media for 24 h and then analyzed for *ALDH1A1* mRNA expression by real time qPCR as in Figure 2A. Relative differences in *ALDH1A1* gene expression for each pair of cells (for each subject under both conditions, vehicle-treated and TNF-treated) were normalized to expression levels of a housekeeping gene, *HPRT1*. **(C)** Enhanced PDPN surface expression by TNF. Healthy control ME-SFCs (*n* = 9) were treated with vehicle (Veh) and TNF (10 ng/ml) for 48 h in separate flasks, and then analyzed for PDPN surface expression by flow cytometry. Data are shown as MFI (corrected for isotype control) for each subject under both conditions (vehicle-treated and TNF-treated). For **(A–C)**, paired data points (after vehicle treatment and TNF treatment) are shown for the cells from each subject. **(A–C)** Significance was determined using paired students *t*-test. *P < 0.05; **P < 0.01; ***P < 0.001. **(D)** Representative histograms of PDPN surface expression after exposure of control ME-SFCs to vehicle and TNF. Healthy control ME-SFCs were treated with vehicle and TNF (1 ng/ml or 10 ng/ml) in separate flasks for 48 h, and then analyzed for PDPN surface expression by flow cytometry. The histogram plot for isotype control, PDPN-vehicle, PDPN-TNF (1 ng/ml), and PDPN-TNF (10 ng/ml) with each mean MFI is shown.

Given the prior evidence for chronic inflammation in the eutopic endometrium in endometriosis, we examined whether the endometriosis-associated ME-SFC phenotypes (impaired decidualization, reduced *ALDH1A1* expression and enhanced cell surface PDPN expression) were induced by chronic exposure of ME-SFCs to TNF. In a paired analysis of ME-SFCs isolated from control subjects, we clearly show a reduction in cAMP-induced decidualization (i.e., IGFBP1 production) on day 7 following TNF exposure on days 1 and 3, when compared to control ME-SFCs exposed to vehicle on the same days (Figure 3A, *P* < 0.01).

Similarly, ME-SFCs from healthy controls exposed to TNF (10 ng/ml) on days 1 and 3 exhibited a marked reduction in *ALDH1A1* mRNA expression (Figure 3B, *P* = 0.029) when analyzed on day 7. Inhibition of *ALDH1A1* expression was observed following chronic IL-1β exposure, as well as lower doses (1 ng/ml) of TNF (Supplemental Figures 1A,B). Likewise, but in a manner opposite to decidualization and *ALDH1A1* expression, surface expression of PDPN was strongly upregulated by prior treatment of control ME-SFCs with TNF (Figure 3C, *P* < 0.001). An example of PDPN data shown as a histogram for a representative control subject's ME-SFCs treated with vehicle or increasing doses of TNF is shown in Figure 3D.

### ME-SFCs Exhibit Additional Phenotypes Relevant to the Pathogenesis of Endometriosis

**Figure 4:**
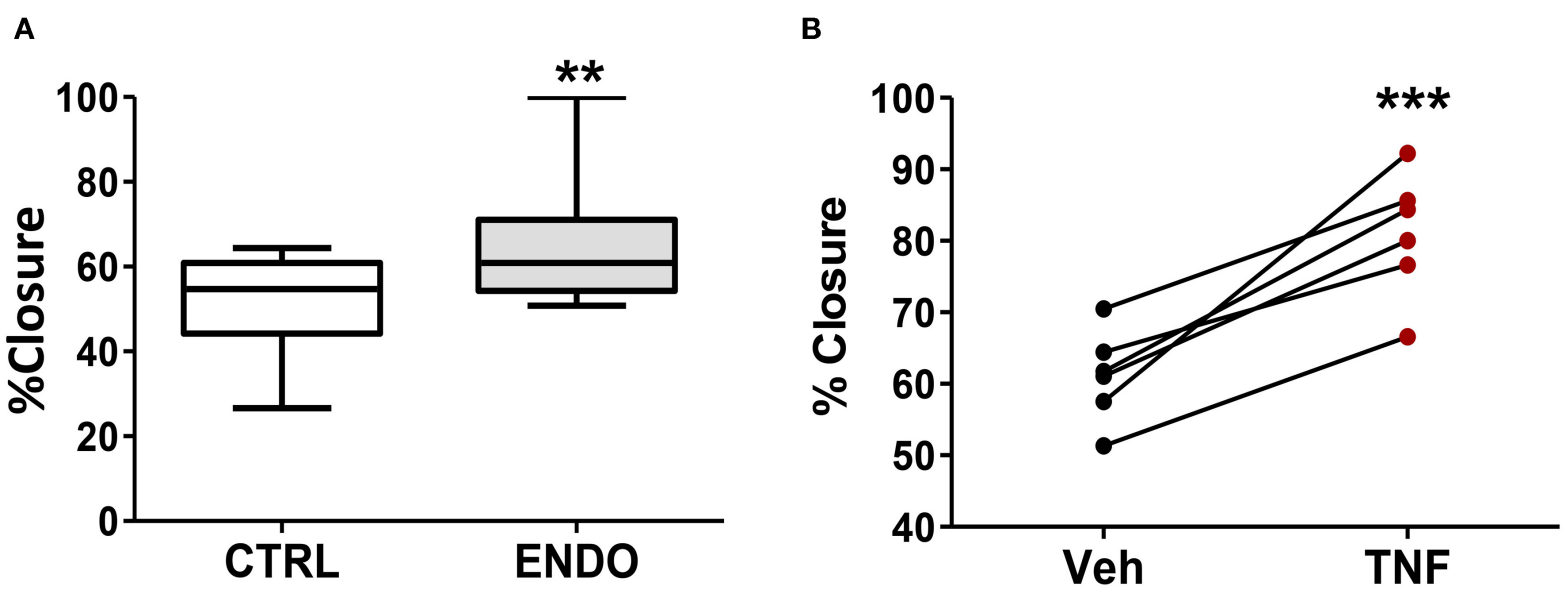
ME-SFCs from endometriosis patients exhibit enhanced cell migration compared to control ME-SFCs; treatment of control ME-SFCs with TNF promotes cell migration. **(A)** Increased cell migration by ME-SFCs from endometriosis patients. Healthy control ME-SFCs (CTRL, *n* = 15) and endometriosis ME-SFCs (ENDO, *n* = 16) were analyzed for cell migration using the scratch assay method as described in the methods section. Monolayers were photographed at time 0 and after 20 h (see Supplemental Figure 2). Images were imported into Image J and cell migration was determined as % wound or scratch closure. Data are shown for ME-SFCs from the controls (white box) and endometriosis subjects (gray box) using Tukey box and whisker plots (box = interquartile range; horizontal line = median; upper and lower whiskers indicate range without outliers; outliers = •). Significance was determined using unpaired students *t*-tests with Welch's correction. **(B)** Increased migration of control ME-SFCs after exposure to TNF. Healthy control ME-SFCs (*n* = 6) were treated with vehicle and TNF (10 ng/ml), in separate flasks, on days 1 and 3. On day 7 cells were analyzed for cell migration using the scratch assay method as described in A. Data are shown as paired data points (after vehicle treatment and TNF treatment) for each healthy control. Significance was determined using a paired students *t*-test **(B)**. **P < 0.01; ***P < 0.001.

Because we observed increased PDPN expression on the surface of ME-SFCs from endometriosis patients vs. controls (Figure 2B), we compared the migration of endometriosis ME-SFCs to control ME-SFCs using the standard scratch-migration assay. Endometriosis ME-SFCs exhibit enhanced migration compared to control ME-SFCs under basal conditions (Figure 4A, *P* < 0.01). Because we observed that PDPN expression can be upregulated on the surface of fibroblasts by TNF (as in Figure 3C), we also compared cell migration of control ME-SFCs after exposure to TNF vs. vehicle. Control ME-SFCs exhibit significantly enhanced migration following TNF treatment (Figure 4B, *P* < 0.001). See Supplemental Figure 2 for representative migration images.

**Figure 5:**
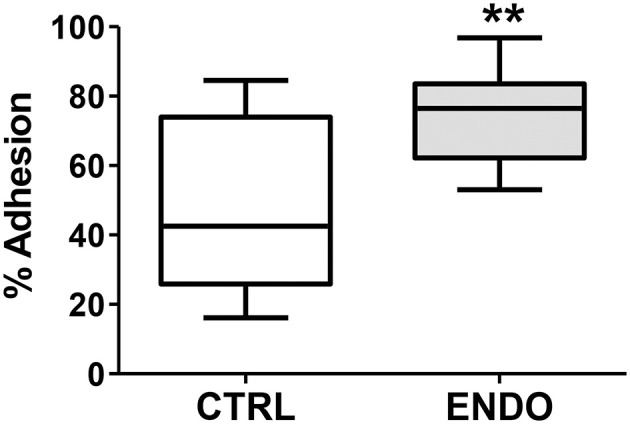
Endometriosis ME-SFCs are more adherent to fibronectin than control ME-SFCs. Increased adherence of ME-SFCs to fibronectin in endometriosis patients. ME-SFCs from healthy controls (CTRL, *n* = 12) and ME-SFCs from endometriosis patients (ENDO, *n* = 9) were analyzed for adherence to fibronectin-coated plates after a 20 min incubation. Non-adherent cells were removed by washing and the % of adherent cells (vs. total cells added) was determined by counting using CyQUANT compared to a standard curve of known cell numbers. Data are shown as the mean % adhesion for the control subjects (white box) and endometriosis subjects (gray box) using Tukey box and whisker plots (box = interquartile range; horizontal line = median; upper and lower whiskers indicate range without outliers). No outliers were observed. Significance was determined using an unpaired *t*-test with Welch's correction **P < 0.01.

In addition to enhanced cell migration, we tested whether ME-SFCs (which would be transported into the peritoneal cavity of control and endometriosis patients) might exhibit more adherence in the endometriosis cases. As shown in Figure 5, ME-SFCs from endometriosis patients display a much higher rate of adhesion to fibronectin when compared to ME-SFCs from control subjects (*P* < 0.01).

### Transient Treatment of Control ME-SFCs With TNF or IL-1β Leads to a Persistent Suppression of Their Decidualization Capacity

**Figure 6:**
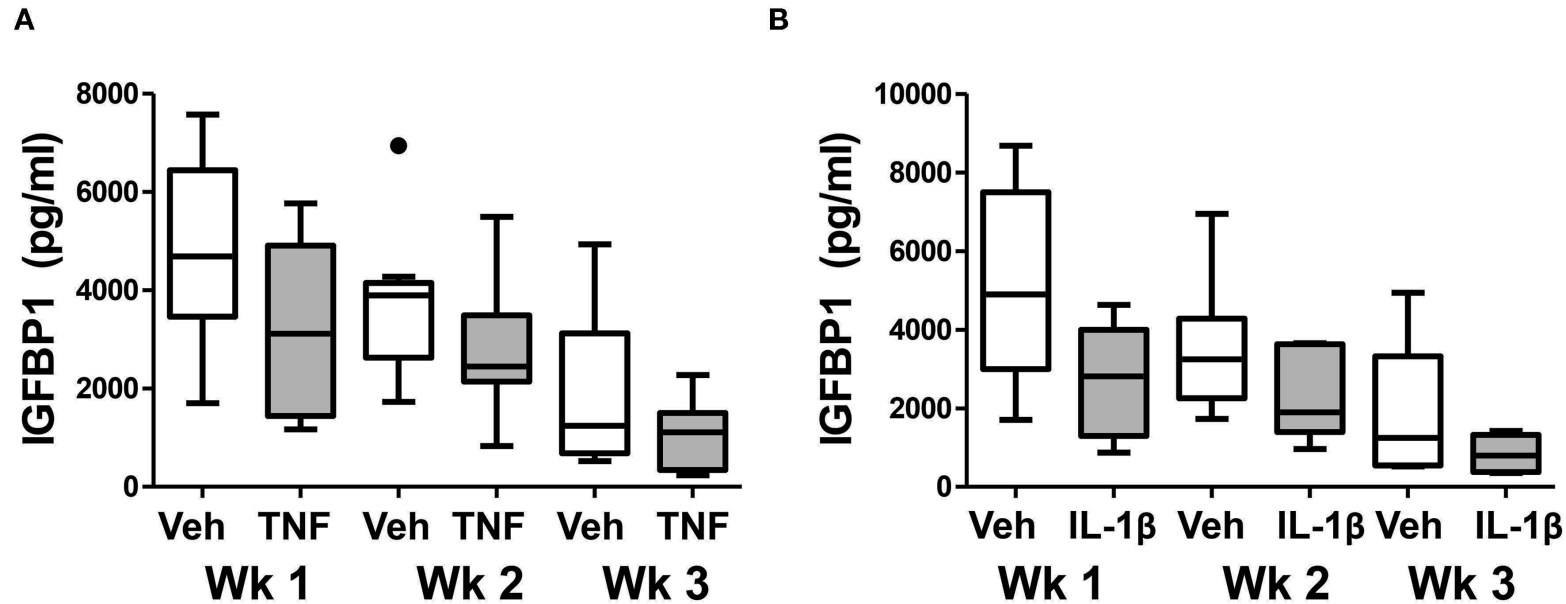
Treatment of control ME-SFCs with cytokines induces persistent changes in decidualization capacity reflecting an endometriosis-like phenotype. Healthy control ME-SFCs (*n* = 9) were treated with **(A)** vehicle (Veh) and TNF (1 ng/ml) or **(B)** vehicle (Veh) and IL-1β (1 ng/ml) (*n* = 8) on days 1 and 3; on day 7 cells were then cultured and passaged in normal growth media. At 1 week (Wk 1), 2 weeks (Wk 2), and 3 weeks (Wk 3) post treatment, each subject's ME-SFCs were assessed for decidualization capacity following treatment with vehicle or cAMP (0.5 mM), as described in Figure 1A. Each subject's data plotted individually are shown in the Supplemental Figure 4. Culture supernatants were analyzed for IGFBP1 (pg/ml) levels by ELISA after 24 hrs. Data are shown as IGFBP1 values for each group's ME-SFCs [vehicle-treated (white box) vs. TNF-treated (gray box), or vehicle-treated (white box) vs. IL-1β-treated (gray box) at weeks 1, 2, or 3 using Tukey box and whisker plots (box = interquartile range; horizontal line = median; upper and lower whiskers indicate range without outliers; outliers = •)]. A persistent reduction in IGFBP1 production over 3 weeks is observed in responses to TNF (***P < 0.001) and IL-1β (**P < 0.01) compared with vehicle as shown in Supplemental Figure 3.

Finally, we examined whether chronic exposure of control ME-SFCs to inflammatory cytokines (e.g., TNF and IL-1β) *ex vivo* would lead to a persistent defect in decidualization. As shown in Figure 6A, the inhibitory effect of prior TNF exposure on decidualization by control ME-SFCs is sustained for 2 and 3 weeks post-TNF exposure. Similar results were observed with IL-1β (Figure 6B). The magnitude of these changes, compared to vehicle alone at baseline, are quite consistent across these time points for both TNF and IL-1β (Supplemental Figures 3A–D). Also, the trends for each subject's ME-SFCs (±TNF or±IL-1β) in the decidualization assay over the 3 week period are shown in Supplemental Figure 4.

## Discussion

Endometriosis is a common, heterogeneous and enigmatic disorder that presents enormous challenges for understanding pathogenesis as well as for diagnosis and clinical management. As summarized in a recent comprehensive review (2), the difficulty and delay in diagnosis of endometriosis is a major barrier to progress and improved patient outcomes, due in part to the lack of a reliable non-invasive diagnostic test, compounded by the lack of awareness, the non-specific nature of symptoms, and the tendency to normalize pain symptoms in these patients. The results in this report indicate that analysis of menstrual effluent (ME) can make a major contribution to solving the diagnostic conundrum of endometriosis and to providing a resource for investigating abnormalities of the endometrium.

Prior studies have demonstrated that stromal cells isolated and cultured from endometrial biopsies of endometriosis patients show defective decidualization when compared to those from healthy controls (33, 34). Decidualization is a process involving the functional and morphological differentiation of endometrial stromal cells that converts them from fibroblast-like cells into larger cuboidal-shaped cells capable of secreting growth factors critical for establishing and maintaining a pregnancy (35). Although the cause of this decidualization defect in endometriosis patients is not-well understood, it has been linked to reduced fertility (36). As shown in Figure 1A, endometriosis cases show a significant decidualization impairment when compared to controls. These data confirm our prior studies showing a similar striking decidualization defect using ME-SFCs collected from a much smaller cohort of diagnosed endometriosis patients and healthy controls (21). Using an ROC curve, defects in the decidualization capacity of ME-SFCs are strongly predictive of the presence of endometriosis (AUC = 0.92) (Figure 1B), with an optimal cut-off point of 14.2 for IGFBP1 by ELISA. We are therefore embarking on a large prospective study of subjects with a symptom complex consistent with endometriosis, who will undergo subsequent endometriosis confirmation by laparoscopy or surgery in order to fully define the sensitivity and specificity of these new diagnostic measures. The similarity in IGFBP1 levels among symptomatic and endometriosis patients (Figure 1A) supports this approach. This is required to transition these promising findings into a clinical test. Clearly, the availability of a reliable non-invasive diagnostic for endometriosis will go a long way toward reducing the 6–10 years diagnostic delay that adolescents and women with endometriosis currently face. However, deficits in decidualization may contribute to other conditions [e.g., uterine infertility [unrelated to endometriosis] (37), and polycystic ovarian syndrome [PCOS] (38)]. Therefore, to optimize accuracy, a diagnostic test for endometriosis based on ME analysis should be carried out in a clinical setting where symptoms of endometriosis are present. In addition, alterations in *ALDH1A1* gene expression and PDPN surface expression may contribute to diagnostic accuracy. While *ALDH1A1* mRNA expression is significantly reduced in endometriosis ME-SFCs (Figure 2A), PDPN surface expression is significantly enhanced in endometriosis ME-SFCs (Figure 2B).

These findings potentially extend beyond the use of *ALDH1A1* and PDPN expression in the diagnosis of endometriosis and provide information about disease pathogenesis. *ALDH1A1* encodes an enzyme that converts retinal into retinoic acid; this pathway has been implicated in endometriosis (39). Retinoic acid is necessary for endometrial cell decidualization (40) and retinoic acid deficiency resulting from impaired retinoic acid biosynthesis has been reported in endometriosis (41). In addition, treatment of endometriosis with retinoic acid analogs has been proposed (42). Increased cell surface podoplanin (PDPN) expression has been described on the surface cancer-associated fibroblast cells and implicated in tumor cell invasiveness (43) and on stromal fibroblasts collected from inflammatory joints of rheumatoid arthritis patients (44, 45). These invasive, pathogenic stromal fibroblasts likely contribute to the tissue invasion and subsequent joint destruction characteristic of this disease. Similarly, PDPN has been identified as a biomarker. Thus, these diagnostic markers may inform us about disease processes.

Our approach and data focused on ME also suggest an aspect of the pathogenic pathway that may provide new opportunities for prevention and treatment. While retrograde menstruation may not be the only mechanism by which endometrial-like tissues are seeded outside of the uterus, studies in menstruating primates and other data in humans strongly support that this model likely holds for many patients, as endometriosis only occurs in menstruating mammals (4). On the other hand, retrograde menstruation is common in most women (46), and therefore additional factors must permit or facilitate the implantation and growth of shed endometrial cells and tissues. Genetic and hormonal factors have been a longstanding focus of research, and the presence and regulation of the inflammatory response within the endometriosis lesions themselves are clearly a potential target for therapy. Our findings emphasize the potential role of intrauterine inflammation in changing the biology of endometrial stromal cells (or other cells) in order to enhance the likelihood of these cells initiating and/or promoting endometriosis lesions after retrograde menstruation. The association between endometriosis and chronic endometritis provides support for this “chronic intrauterine inflammation” hypothesis (17, 18). Chronic endometritis is found in endometriosis patients at a much higher rate than controls, 42 vs. 15% in one study (17), and 52 vs. 27% in a second study (18). Chronic endometritis is a poorly characterized clinical entity that is often mildly symptomatic and not typically diagnosed except in the setting of infertility. Although no universally accepted criteria exists, its current diagnosis relies on an endometrial biopsy exhibiting increased numbers of plasma cells in the stromal cell area (22). Bacterial pathogens commonly found in the vaginal tract and cervix are proposed to be the leading cause of chronic endometritis, as it is often successfully treated with antibiotics (22). Endometrial inflammation also can be caused by sexually transmitted infections (e.g., chlamydia and gonorrhea) (47). Interestingly, pelvic inflammatory disease, believed to be mainly caused by *Mycoplasma genitalium*, is also associated with increased endometrial inflammation, infertility and endometriosis (48, 49). Regardless of the cause, an increase in inflammatory cytokines and immune cells have been found in the endometrial tissue and ME from patients with endometritis, chronic endometritis and pelvic inflammatory disease (22, 23, 49). While direct causation has not been established, the fact that endometriosis has been associated with chronic endometritis on biopsy lends support to the hypothesis that chronic inflammation of the eutopic endometrium may predispose to the development of endometriosis, through changes in the biology of the endometrium that is subsequently shed into the pelvic cavity at menstruation (17, 18).

A striking aspect of the phenotypic changes we have observed with ME-SFCs is that these changes are maintained after weeks of culture from freshly obtained menstrual effluent, with no difference in the culture conditions for ME-SFCs from controls and endometriosis patients. Previous studies report that the eutopic endometrium of patients with endometriosis is in fact different from the eutopic endometrium of controls, with changes in gene expression, and the presence of inflammatory mediators, including TNF (12–14). Several cell types in the endometrium produce TNF, including stromal fibroblasts and epithelial cells (50–52). Similarly, cultured stromal cells from endometrial and endometriosis lesions express TNF *ex vivo* (53, 54). TNF mediates its effects by binding to membrane TNF receptors [e.g., TNF receptor (TNFR)1 and 2] (55, 56), which are expressed by numerous cells in the endometrium, including stromal cells (57). We show that exposure of ME-SFCs from control subjects to TNF replicates the phenotypes we observed in ME-SFCs from endometriosis patients, including reduced decidualization (Figure 3A), reduced expression of *ALDH1A1* (Figure 3B), increased expression of PDPN (Figure 3C), and enhanced migration capacity (Figure 4B). The fact that we were able to observe persistent defects in decidualization capacity several weeks after *in vitro* exposure of normal ME-SFCs to TNF or IL-1β (see Figure 6 and Supplemental Figures 3, 4) supports the concept that a similar exposure to inflammation in the eutopic endometrium explains the persistence of these phenotypes in ME-SFCs derived from fresh ME.

This model of endometriosis pathogenesis driven by chronic intrauterine inflammation suggests numerous therapeutic approaches. It is likely that recurrent retrograde menstruation contributes to the progression of endometriosis, with continued seeding of the peritoneal cavity. Therefore, anti-inflammatory therapies including cytokine blockade may reverse the stromal phenotypes that predispose to implantation, as well as treat the inflammatory process in established lesions. Several studies in non-human primates support early anti-TNF therapy. In the baboon model, treatment with anti-TNF antibodies (58) and soluble TNFR1 significantly reduced the induction and progression of endometriosis (59). Although TNF blockade has not proved to be effective in mitigating endometriosis pain in women (60), to our knowledge, the effect of TNF blockade on endometriosis disease progression in humans has not been examined and no clinical trials of cytokine blockade have been carried out in endometriosis patients with early disease. However, trials of IL-1β blockade are reportedly in the planning stages [Reviewed in (2)].

We have shown enhanced migration (Figure 4A) and adhesion to a fibronectin substrate by ME-SFCs from patients with endometriosis when compared to control ME-SFCs (Figure 5). It is possible that the increased expression of PDPN on ME-SFCs (Figure 2B) may contribute to these phenotypes, although we have not yet directly demonstrated this. PDPN expression on cancer-associated fibroblasts (CAFs) is regulated by inflammatory cytokines within tumors and is associated with enhanced tumor invasion and has emerged as a target for cancer therapy (61). Similarly, PDPN expressed on the surface of pathogenic stromal fibroblasts found in arthritic joints has been proposed to be a therapeutic target for rheumatoid arthritis (62). To our knowledge, PDPN protein expression in endometriosis lesions has not yet been investigated.

There are several limitations of this study. First, the control group is comprised of healthy controls who self-report their lack of endometriosis symptoms (i.e., they have not been surgically verified as controls) and therefore, the control group may contain subjects with unknown endometriosis. However, this would be less or equal to 4% based on the prevalence of asymptomatic endometriosis in a “general population” of women undergoing tubal ligation (63). By contrast, all endometriosis cases in this study were surgically and histologically confirmed. Another limitation is that the symptomatic group shown in Figure 1A has not yet been surgically diagnosed. Other limitations of this study are the limited sample sizes and unequal sample sizes for some of the cell phenotyping and functions assays. However, the main predictor (decidualization capacity) has relatively large numbers of cases and controls and all experimental comparisons for all phenotypes and functional assays employed age-matched cells. In addition, all experiments were performed using subjects' cells of the same passage number in order to achieve consistent results. In the future it will be important to show that ME-derived cells collected non-invasively at different monthly cycles show similar results in the same women. Finally, although our hypothesis that chronic intrauterine inflammation is present in endometriosis is strongly supported by the literature, we were unable to directly assess this in either cases or controls *in vivo*.

In conclusion, we demonstrate that ME-derived stromal cells can be leveraged to develop a non-invasive diagnostic for endometriosis, a condition with a known 6–10 years delay in diagnosis and reliance on invasive surgical diagnosis. In addition, our studies show that ME provides a biologic tool to study the pathogenesis of endometriosis and may eventually lead to novel, more effective treatments. Because ME sampling is non-invasive, it can be used for large population studies to identify genetically driven phenotypes such as expression quantitative trait loci or eQTLs in the many regions that have been defined in GWAS studies (2). Thus, ME is likely to provide an important resource for functional population studies of the complex genetics underlying endometriosis.

## Data Availability Statement

All datasets generated for this study are included in the article/Supplemental Material. Raw data are available on request to the corresponding authors.

## Ethics Statement

The studies involving human participants were reviewed and approved by The Human Research Protection Program/Institutional Review Board (IRB) of Northwell Health (IRB #13-376A and IRB #13-627A). The patients/participants provided their written informed consent to participate in this study.

## Author Contributions

CM, PG, MY, and AN: conceptualization. CM, PG, MY, AN, MS, AS, PC, XX, and RV: methodology. AN, MS, XX, PC, and RV: validation. AS, CM, PG, and AN: format analysis. CM, PG, MY, AN, MS, PC, and XX: investigation. MD, KE, GK, and EK: resources. EK, KE, MD, GK, AN, MS, CM, and PG: data curation. CM, PG, and AN: writing—original draft and supervision. CM, PG, AN, and MS: writing—reviewing and editing. CM, PG, AN, MS, PC, and XX: visualization. CM and PG: project administration. PG and CM: funding acquisition. All authors reviewed final manuscript.

## Conflict of Interest

PG and CM are names as inventors on a patent application describing the use of menstrual effluent for the diagnosis of endometriosis. The remaining authors declare that the research was conducted in the absence of any commercial or financial relationships that could be construed as a potential conflict of interest.

## Supplementary Material

Supplemental Figure 1 ALDH1A1 gene expression is reduced following treatment of control ME-SFCs with TNF and IL-1β. **(A)** A reduction in *ALDH1A1* mRNA expression is observed after treatment with TNF and IL-1β vs. vehicle. Healthy control ME-SFCs (*n* = 19) were treated with vehicle (Veh), TNF (1 ng/ml), and IL-1β (1 ng/ml), in separate flasks, on days 1 and 3. On day 7 cells were lifted, washed and plated in decidualization media for 24 h and then analyzed for *ALDH1A1* mRNA expression by real time qPCR as in Figure 2A. All samples were run in triplicate. Relative differences in *ALDH1A1* gene expression [for each subject under all conditions, vehicle, TNF and IL-1β] were normalized to expression levels of a housekeeping gene, *HPRT1*. **(B)** Dose response reduction in *ALDH1A1* gene expression following treatment with vehicle or TNF (1 and 10 ng/ml). Healthy control ME-SFCs (*n* = 19) were treated with vehicle (Veh) or TNF (1 and 10 ng/ml), in separate flasks, on days 1 and 3. On day 7 cells were lifted, washed and plated in decidualization media for 24 h and then analyzed for *ALDH1A1* mRNA expression by real time qPCR as in Figure 2A. All samples were run in triplicate. Relative differences in *ALDH1A1* gene expression [for each subject under all conditions, vehicle, TNF and two doses of TNF (1 and 10 ng/ml)] were normalized to expression levels of a housekeeping gene, *HPRT1*. Data are shown as relative ALDH1A1 mRNA expression using Tukey box and whisker plots (box = interquartile range; horizontal line = median; upper and lower whiskers indicate range without outliers; outliers = •). Significance was determined using the Kruskal-Wallis test followed by the Dunn's test for multiple comparisons. ^**^*P* < 0.001; ^***^*P* < 0.0001.

Supplemental Figure 2 Endometriosis ME-SFCs show increased cell migration; treatment of control ME-SFCs with TNF enhances migration. Representative images for cell migration assays; images were captured at time 0 (immediately after scratch) and 20 h after the scratch. ME-SFCs from two control (CTRL) individuals treated with vehicle alone are shown in the top row. ME-SFCs from two patients with endometriosis (ENDO) treated with vehicle alone are shown in the middle row. ME-SFC from two control individuals after prior exposure to TNF (CTRL+TNF) are shown in the bottom row. Yellow lines outline the outer edges of the scratch at each time point.

Supplemental Figure 3 Cytokine treatment of ME-SFCs leads to sustained defects in decidualization. **(A)** Inhibition of IGFBP1 production by TNF at 1, 2, and 3 weeks post-TNF treatment. Healthy control ME-SFCs (*n* = 9) were treated with vehicle (Veh) and TNF (10 ng/ml) in separate flasks on days 1 and 3. On day 7 ME-SFCs were lifted, washed and plated in decidualization media. Decidualization capacity was analyzed after week 1 (Wk 1), week 2 (Wk 2), and week 3 (Wk 3) post-treatment after lifting and treating the vehicle- and TNF-treated cells with vehicle and cAMP (0.5 mM), as described in Figure 1A. All samples were run in triplicate. After 24 h culture supernatants of vehicle- and TNF-treated ME-SFCs at each time point were analyzed for IGFBP1 concentrations by ELISA. Using natural log-transformed data the inhibition of decidualization by TNF at each week post-treatment was determined for each subject as the natural logarithm or ln (TNF/vehicle). **(B)** Average inhibition of decidualization by TNF over weeks 1 through 3. Using the data in Figure 6 showing there is no difference in TNF-reduced decidualization over the 3 weeks (i.e., inhibition was sustained), the average inhibition by TNF for each subject's cells across the 3 weeks was averaged. **(C)** Inhibition of IGFBP1 by IL-1β at 1, 2, and 3 weeks post-IL-1β treatment. Healthy control ME-SFCs (*n* = 8) were treated with vehicle (Veh) and IL-1β (1 ng/ml) in separate flasks on days 1 and 3. On day 7 ME-SFCs were lifted, washed and plated in decidualization media. Decidualization capacity was analyzed after week (Wk1, Wk2, and Wk3) after lifting and treating the vehicle- and IL-1β-treated cells with vehicle and cAMP (0.5 mM), as described in Figure 6. All samples were run in triplicate. After 24 h cell-free culture supernatants of vehicle- and IL-1β-treated ME-SFCs at each time point were analyzed for IGFBP1 concentrations by ELISA. Using natural log-transformed data the inhibition of decidualization by IL-1β at each wk post-treatment was determined for each subject as the natural logarithm or ln (IL-1β/vehicle). **(D)** Average inhibition of decidualization by IL-1β at weeks 1, 2, and 3 post treatment. Using the data in Figure 6 showing there is no difference in IL-1β-reduced decidualization over the 3 weeks (i.e., inhibition was sustained), the average inhibition by IL-1β for each subject's cells across the 3 weeks was averaged. Data points are shown for each subject's ME-SFCs, with mean ± SEM at each time point (*n* = the number of subjects per group). Significance was determined using repeated measures ANOVA (using natural log-transformed data; TNF/Vehicle or IL-1β/Vehicle) to analyze the effect of TNF or IL-1β on decidualization over weeks 1–3. Once we demonstrated that the patterns were the same across time (weeks 1–3), i.e., there was no difference in the effect of cytokine-induced decidualization inhibition over time **(A,C)**, then the means at each wk were pooled and a one sample *t*-test was used for comparison **(B,D)**. ^**^*P* < 0.01 and ^***^*P* < 0.001.

Supplemental Figure 4 Treatment of control ME-SFCs with cytokines induces persistent changes in decidualization capacity reflecting an endometriosis-like phenotype: individual data. Healthy control ME-SFCs (*n* = 9) were treated with **(A)** vehicle (Veh) and TNF (1 ng/ml) or **(B)** vehicle (Veh) and IL-1β (1 ng/ml) (*n* = 8) on days 1 and 3; on day 7 cells were then cultured and passaged in normal growth media. At 1 week (Wk 1), 2 weeks (Wk 2), and 3 weeks (Wk 3) post treatment, each subject's ME-SFCs were assessed for decidualization capacity following treatment with vehicle or cAMP (0.5 mM), as described in Figure 6 (showing aggregate data). Decidualization data are shown for each individual's ME-SFCs plotted at week 1, week 2, and week 3 for ±TNF-treated cells **(A)** and ±IL-1β-treated cells **(B)**.

## Abbreviations

ALDH1A1: Aldehyde dehydrogenase 1 family member A1 gene
AUC: area under the curve
cAMP: dibutyryladenosine- 3′, 5′- cyclic monophosphate
ENDO: endometriosis
FBS: fetal bovine serum
IGFBP1: insulin growth factor binding protein 1
IL-1β: interleukin-1β
MALS: *Maackia amurensis*
ME: menstrual effluent
ME-SFCs: menstrual effluent-derived stromal fibroblast cells
PDPN: podoplanin
ROC: receiver operating characteristic
ROC: receiver operating characteristic
SFCs: stromal fibroblast cells
TNF: tumor necrosis factor.
