# Supplementary figures and images for: Menstrual Effluent Provides a Novel Diagnostic Window on the Pathogenesis of Endometriosis
Source: Front Reprod Health. 2020 Jul 22;2:3. doi: 10.3389/frph.2020.00003 (PMC9580670; doi:10.3389/frph.2020.00003)

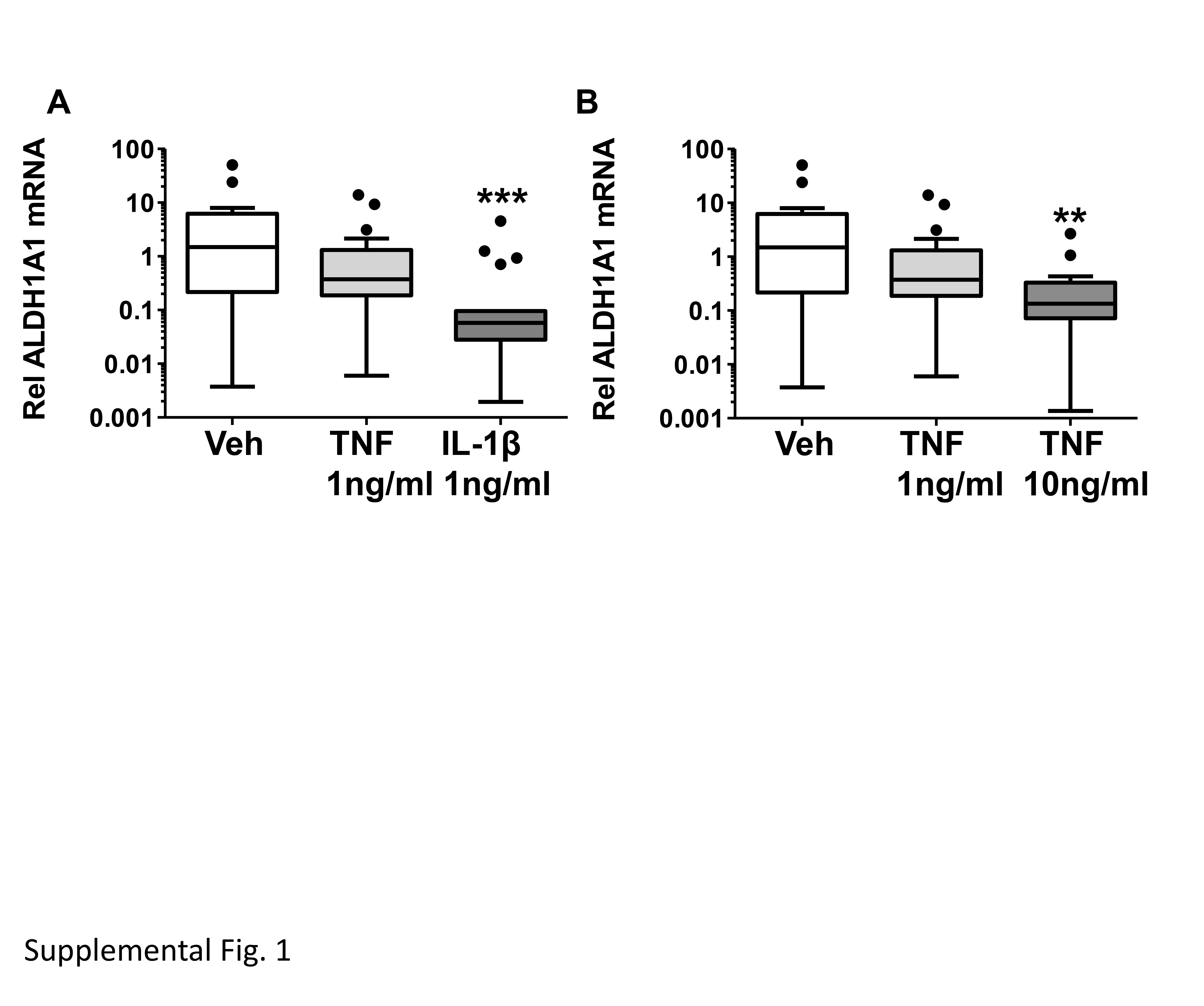

Supplement: Supplemental Figure 1 — ALDH1A1 gene expression is reduced following treatment of control ME-SFCs with TNF and IL-1β. (A) A reduction in ALDH1A1 mRNA expression is observed after treatment with TNF and IL-1β vs. vehicle. Healthy control ME-SFCs (n = 19) were treated with vehicle (Veh), TNF (1 ng/ml), and IL-1β (1 ng/ml), in separate flasks, on days 1 and 3. On day 7 cells were lifted, washed and plated in decidualization media for 24 h and then analyzed for ALDH1A1 mRNA expression by real time qPCR as in Figure 2A. All samples were run in triplicate. Relative differences in ALDH1A1 gene expression [for each subject under all conditions, vehicle, TNF and IL-1β] were normalized to expression levels of a housekeeping gene, HPRT1. (B) Dose response reduction in ALDH1A1 gene expression following treatment with vehicle or TNF (1 and 10 ng/ml). Healthy control ME-SFCs (n = 19) were treated with vehicle (Veh) or TNF (1 and 10 ng/ml), in separate flasks, on days 1 and 3. On day 7 cells were lifted, washed and plated in decidualization media for 24 h and then analyzed for ALDH1A1 mRNA expression by real time qPCR as in Figure 2A. All samples were run in triplicate. Relative differences in ALDH1A1 gene expression [for each subject under all conditions, vehicle, TNF and two doses of TNF (1 and 10 ng/ml)] were normalized to expression levels of a housekeeping gene, HPRT1. Data are shown as relative ALDH1A1 mRNA expression using Tukey box and whisker plots (box = interquartile range; horizontal line = median; upper and lower whiskers indicate range without outliers; outliers = •). Significance was determined using the Kruskal-Wallis test followed by the Dunn's test for multiple comparisons. **P < 0.001; ***P < 0.0001. [file Image_1.TIF]

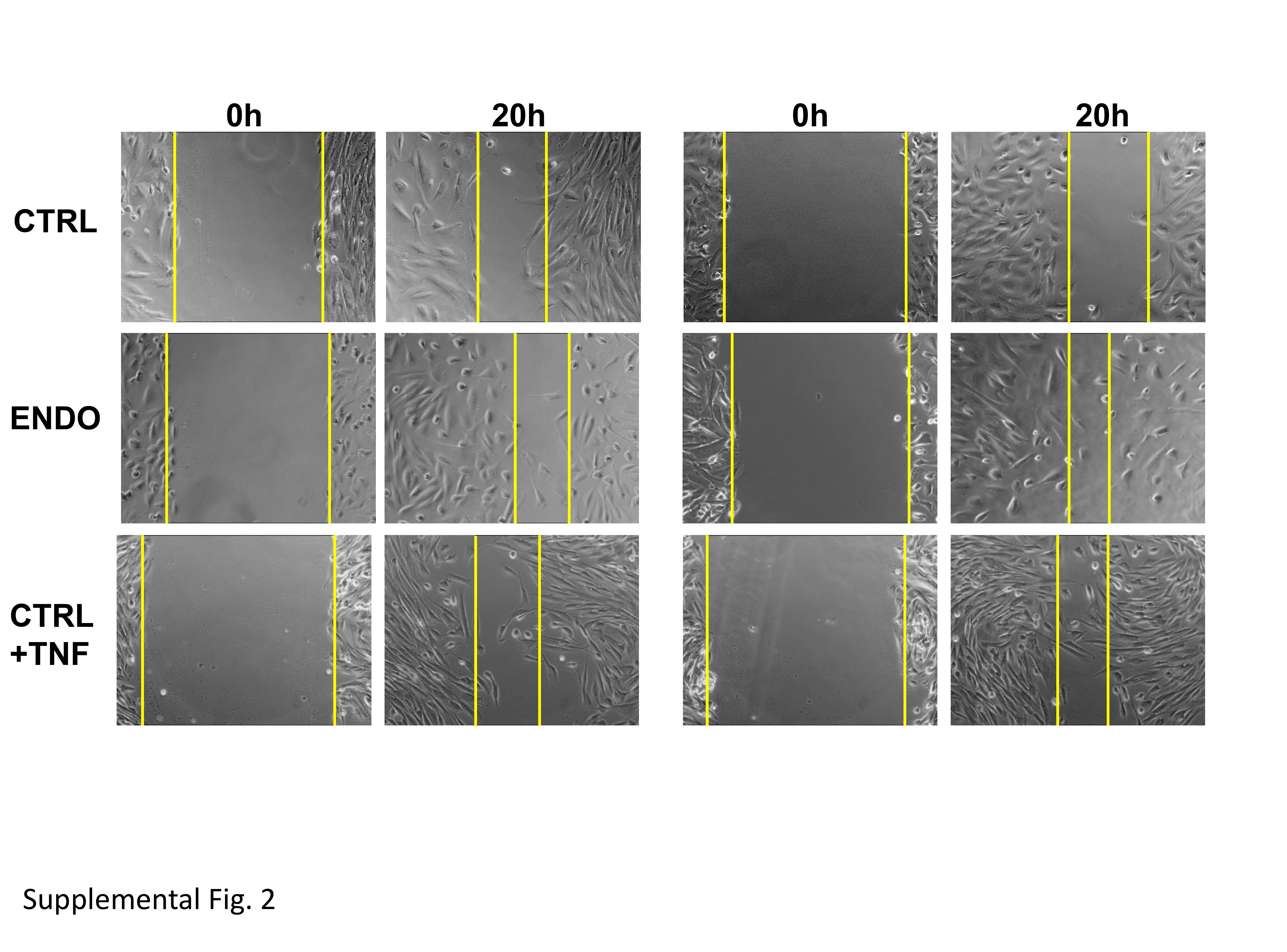

Supplement: Supplemental Figure 2 — Endometriosis ME-SFCs show increased cell migration; treatment of control ME-SFCs with TNF enhances migration. Representative images for cell migration assays; images were captured at time 0 (immediately after scratch) and 20 h after the scratch. ME-SFCs from two control (CTRL) individuals treated with vehicle alone are shown in the top row. ME-SFCs from two patients with endometriosis (ENDO) treated with vehicle alone are shown in the middle row. ME-SFC from two control individuals after prior exposure to TNF (CTRL+TNF) are shown in the bottom row. Yellow lines outline the outer edges of the scratch at each time point. [file Image_2.JPEG]

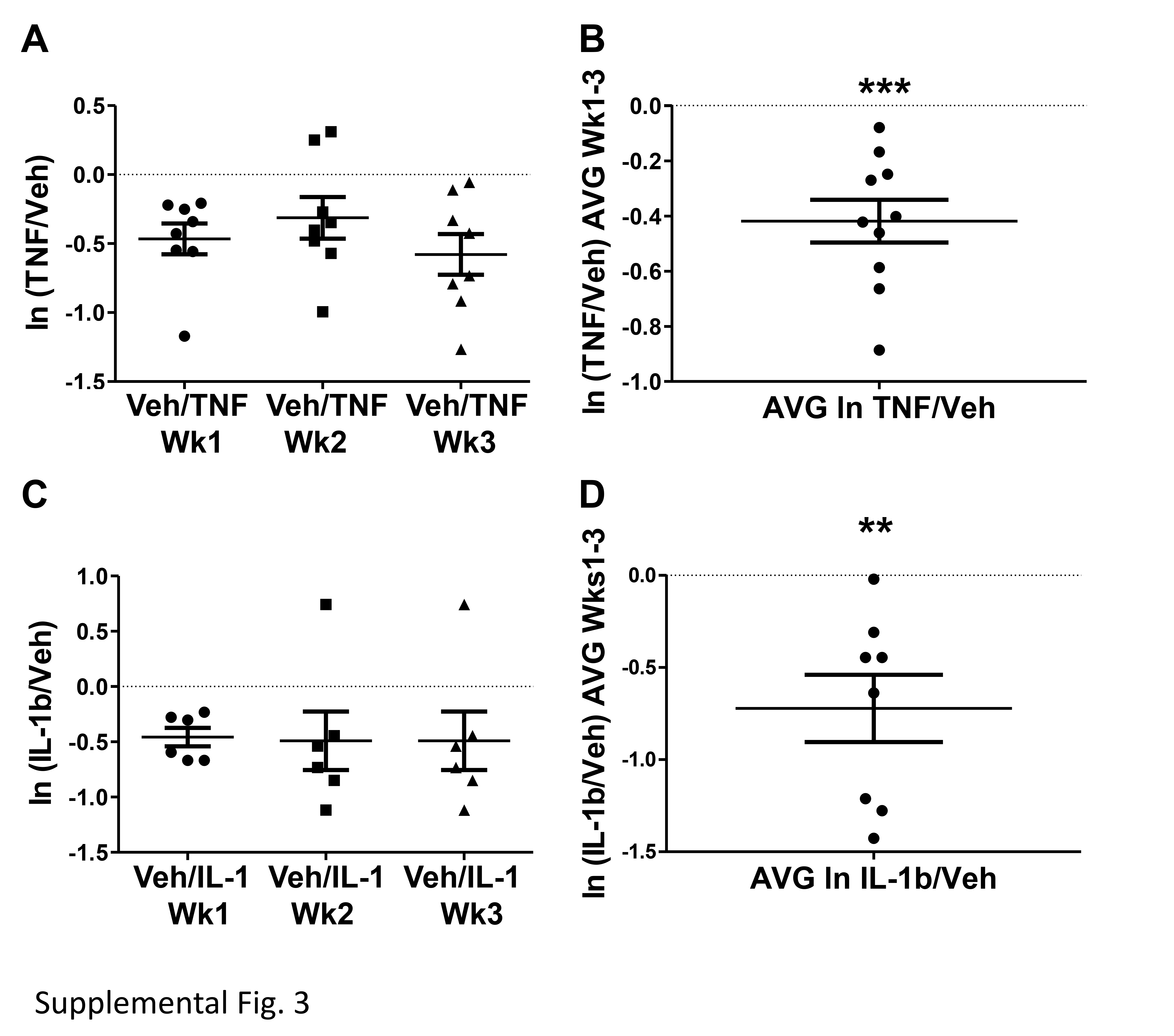

Supplement: Supplemental Figure 3 — Cytokine treatment of ME-SFCs leads to sustained defects in decidualization. (A) Inhibition of IGFBP1 production by TNF at 1, 2, and 3 weeks post-TNF treatment. Healthy control ME-SFCs (n = 9) were treated with vehicle (Veh) and TNF (10 ng/ml) in separate flasks on days 1 and 3. On day 7 ME-SFCs were lifted, washed and plated in decidualization media. Decidualization capacity was analyzed after week 1 (Wk 1), week 2 (Wk 2), and week 3 (Wk 3) post-treatment after lifting and treating the vehicle- and TNF-treated cells with vehicle and cAMP (0.5 mM), as described in Figure 1A. All samples were run in triplicate. After 24 h culture supernatants of vehicle- and TNF-treated ME-SFCs at each time point were analyzed for IGFBP1 concentrations by ELISA. Using natural log-transformed data the inhibition of decidualization by TNF at each week post-treatment was determined for each subject as the natural logarithm or ln (TNF/vehicle). (B) Average inhibition of decidualization by TNF over weeks 1 through 3. Using the data in Figure 6 showing there is no difference in TNF-reduced decidualization over the 3 weeks (i.e., inhibition was sustained), the average inhibition by TNF for each subject's cells across the 3 weeks was averaged. (C) Inhibition of IGFBP1 by IL-1β at 1, 2, and 3 weeks post-IL-1β treatment. Healthy control ME-SFCs (n = 8) were treated with vehicle (Veh) and IL-1β (1 ng/ml) in separate flasks on days 1 and 3. On day 7 ME-SFCs were lifted, washed and plated in decidualization media. Decidualization capacity was analyzed after week (Wk1, Wk2, and Wk3) after lifting and treating the vehicle- and IL-1β-treated cells with vehicle and cAMP (0.5 mM), as described in Figure 6. All samples were run in triplicate. After 24 h cell-free culture supernatants of vehicle- and IL-1β-treated ME-SFCs at each time point were analyzed for IGFBP1 concentrations by ELISA. Using natural log-transformed data the inhibition of decidualization by IL-1β at each wk post-treatment was determi [file Image_3.TIF]

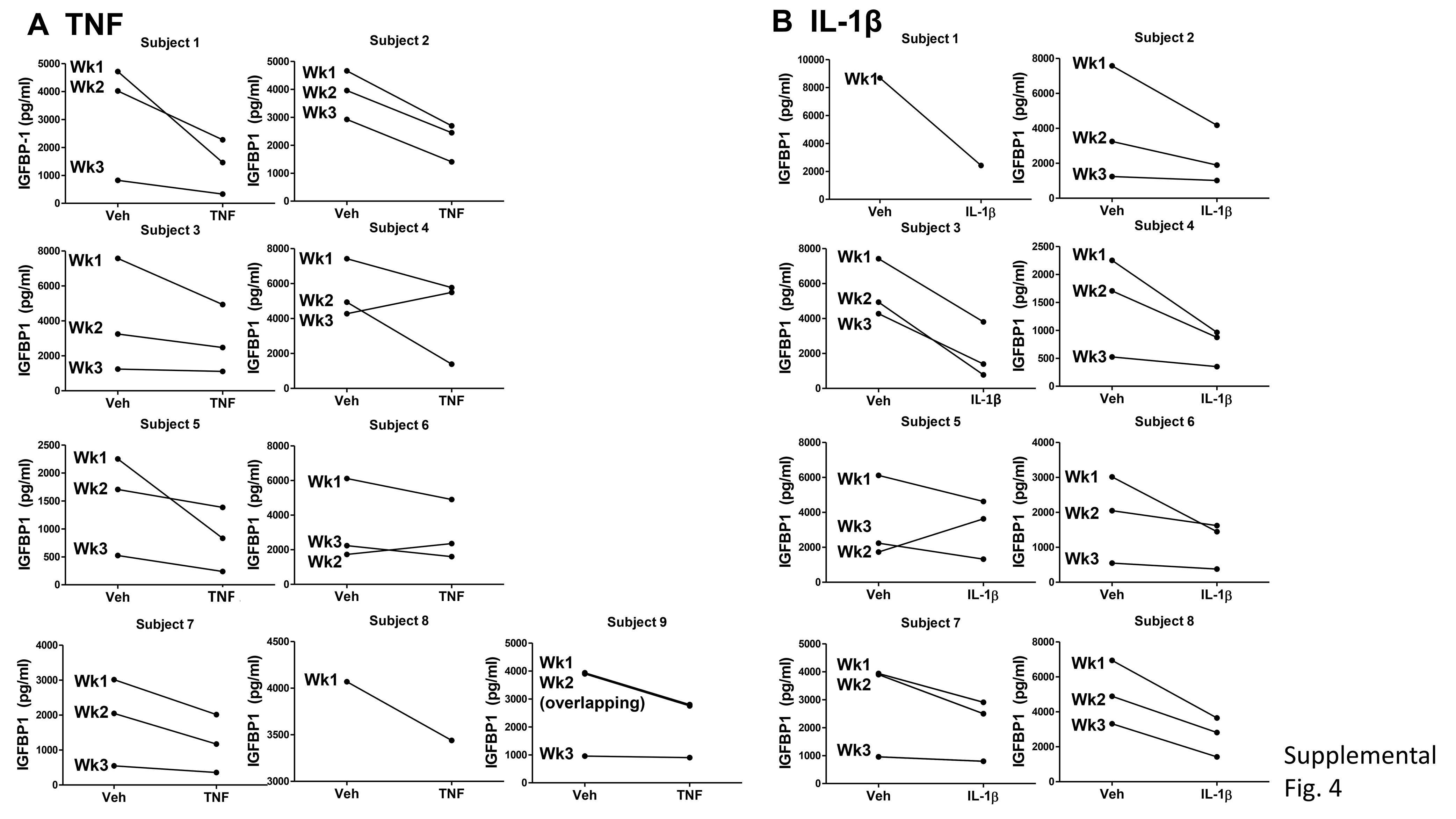

Supplement: Supplemental Figure 4 — Treatment of control ME-SFCs with cytokines induces persistent changes in decidualization capacity reflecting an endometriosis-like phenotype: individual data. Healthy control ME-SFCs (n = 9) were treated with (A) vehicle (Veh) and TNF (1 ng/ml) or (B) vehicle (Veh) and IL-1β (1 ng/ml) (n = 8) on days 1 and 3; on day 7 cells were then cultured and passaged in normal growth media. At 1 week (Wk 1), 2 weeks (Wk 2), and 3 weeks (Wk 3) post treatment, each subject's ME-SFCs were assessed for decidualization capacity following treatment with vehicle or cAMP (0.5 mM), as described in Figure 6 (showing aggregate data). Decidualization data are shown for each individual's ME-SFCs plotted at week 1, week 2, and week 3 for ±TNF-treated cells (A) and ±IL-1β-treated cells (B). [file Image_4.tif]
